# Comparison of SF_6_ and CF_4_ Plasma Treatment for Surface Hydrophobization of PET Polymer

**DOI:** 10.3390/ma11020311

**Published:** 2018-02-21

**Authors:** Matic Resnik, Rok Zaplotnik, Miran Mozetic, Alenka Vesel

**Affiliations:** 1 Jozef Stefan International Postgraduate School, Jamova 39, Ljubljana 1000, Slovenia; matic.resnik@ijs.si; 2 Jozef Stefan Institute, Jamova 39, Ljubljana 1000, Slovenia; rok.zaplotnik@ijs.si (R.Z.); miran.mozetic@ijs.si (M.M.)

**Keywords:** sulphur hexafluoride (SF_6_) plasma, tetrafluoromethane (CF_4_) plasma, polymer polyethylene terephthalate (PET), surface modification, functionalization and wettability, optical emission spectroscopy (OES), electronegativity

## Abstract

The fluorination of the polymer polyethylene terephthalate in plasma created from SF_6_ or CF_4_ gas at various pressures was investigated. The surface was analysed by X-ray photoelectron spectroscopy and water contact angle measurements, whereas the plasma was characterized by optical emission spectroscopy. The extent of the polymer surface fluorination was dependent on the pressure. Up to a threshold pressure, the amount of fluorine on the polymer surface and the surface hydrophobicity were similar, which was explained by the full dissociation of the SF_6_ and CF_4_ gases, leading to high concentrations of fluorine radicals in the plasma and thus causing the saturation of the polymer surface with fluorine functional groups. Above the threshold pressure, the amount of fluorine on the polymer surface significantly decreased, whereas the oxygen concentration increased, leading to the formation of the hydrophilic surface. This effect, which was more pronounced for the SF_6_ plasma, was explained by the electronegativity of both gases.

## 1. Introduction

Fluorine-containing plasmas are often used for the surface hydrophobization of polymer materials [1,2,3,4,5,6,7,8] and for dry-etching in the semiconducting industry [9,10,11,12]. In the latter, the addition of oxygen is used to enhance the etching rate [11]. If no oxygen is added, etching of the surface could be done by using substrate biasing. However, high ion energies can cause sample graphitization [10]. When fluorine plasmas are used to enhance the surface hydrophobicity, two effects can be obtained, namely, functionalization or deposition (polymerization of fluorocarbons), depending on the F/C ratio [13,14]. If the F/C ratio is high (F/C > 3), there is no polymerization, whereas if the F/C ratio is low (F/C ≤ 2), fluorocarbons will polymerize on the surface. Thus, gases such as CF_4_, SF_6_ and C_2_F_6_ do not cause polymerization [14,15] unless CH_4_ is added to change the F/C ratio [16]. CF_4_ is therefore often used for polymer surface modification to introduce nonpolar functional groups. SF_6_ is rarely used, and therefore, literature is scarce. SF_6_ plasma has been used to treat polyethylene terephthalate PET (fabric [1], fibres [7] or film [5]), cotton fibres [7], polypropylene (PP) [3,4], polyethylene (PE) [5], polyvinyl chloride (PVC) [5] and polymethyl methacrylate [2]. The authors reported increased hydrophobicity; however, different authors reported different stabilities of the hydrophobic surface. Selli et al. found that repeated SF_6_ treatment caused more stable hydrophobicity [7]. Walton et al. found a negligible ageing effect after one year for the sample treated for the longest treatment time of 60 s, but this was not the case for the samples treated for shorter times [6]. Mrad et al. observed the ageing of PET, whereas PVC was stable even 210 days after treatment [5]. Polyethylene was quite stable as well, because the contact angle did not change in the first 40 days of ageing, whereas later it slightly decreased. Here, it is worth mentioning that all authors observed fluorine at the surface treated in SF_6_ plasma; however, few authors observed sulphur because of grafting of SF*_x_* species on the treated surface, which were very sensitive to ablation [8,17].

There have also been some reports in the literature on using O_2_ plasma followed by SF_6_. Mangindaan et al. prepared gradient PP surfaces with wettability between 20° and 135° by applying an O_2_ pretreatment followed by SF_6_ plasma treatment under a specially designed mask with an open end and a closed end, which allowed the diffusion of reactive fluorine species [3,4]. The highest fluorine content of 44 at % was found at the open end, and only 3 at % was found at the close end of the mask. In contrast, the oxygen concentration was approximately 11 and 30 at % at the open and closed ends, respectively. The sulphur content was very small at approximately 0.4 at %. The authors also studied the adhesion of fibroblast cells and found that the number of cells decreased from the hydrophobic surface at the open end to the hydrophilic surface at the closed end. Consecutive O_2_ and SF_6_ plasma treatments were also applied by Bi et al. for treatment of Parylene-C to obtain a superhydrophobic surface [18]. Oxygen plasma treatment time was varied, whereas the treatment time in the SF_6_ plasma was kept constant. The hydrophobicity increased with increasing pretreatment time in the O_2_ plasma until saturation was achieved with a contact angle of 169°. The obtained superhydrophobic surface was a result of the increased surface nanoroughness induced by O_2_ plasma treatment, followed by surface fluorination with SF_6_ plasma treatment.

In this paper, we investigated and compared the SF_6_ and CF_4_ plasma created at various pressures on the surface modification of PET polymer films.

## 2. Materials and Methods

### 2.1. Plasma Treatment

**Figure 1:**
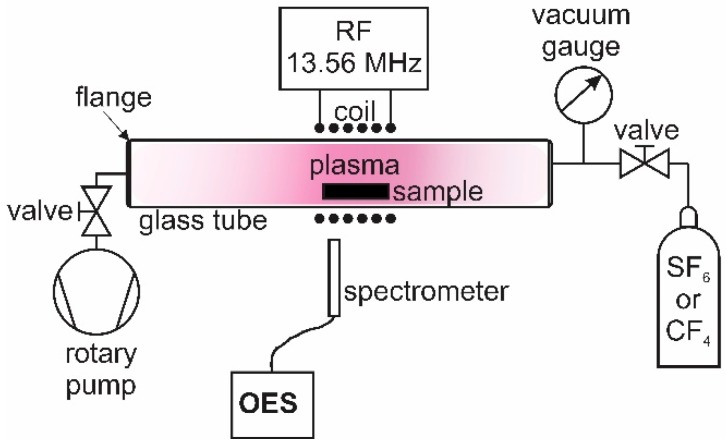
Schematic diagram of the plasma system for sample treatment.

A semi-crystalline PET polymer with a thickness of 0.250 mm was obtained from Goodfellow (Goodfellow Cambridge Ltd., Huntingdon, England). It was cut into small samples of 1 × 1 cm^2^. The samples were treated in a plasma system, as shown in Figure 1. The plasma was created in a Pyrex discharge tube with a length of 80 cm and a diameter of 4 cm. A coil with 6 turns was placed in the centre of the tube. The coil was connected to a radiofrequency RF generator (13.56 MHz) via a matching network. The generator nominal power was fixed to 200 W. The discharge chamber was pumped with a rotary pump with a nominal pumping speed of 80 m^3^·h^−1^. The base pressure was 1 Pa. Sulphur hexafluoride (SF_6_) or tetrafluoromethane (CF_4_) gas (supplied by Messer, Messer Group GmbH, Bad Soden, Germany) was leaked into the plasma chamber, and the gas purity was 99.998 and 99.995, respectively. Samples of the PET polymer were placed in the middle of the coil and treated by plasma at various gas pressures. The lowest pressure was set at 10 Pa, whereas the maximum pressure was determined as a pressure at which it was still possible to ignite the plasma. For the SF_6_ plasma, the highest pressure was 200 Pa, whereas for the CF_4_ plasma, it was 500 Pa. The treatment time was kept constant at 40 s.

### 2.2. Plasma Characterization

The plasma was characterized using optical emission spectroscopy (OES). OES measurements were performed in a quartz tube with a 16-bit Avantes AvaSpec 3648 fibre optic spectrometer (Avantes Inc., Louisville, CO, USA). A nominal spectral resolution was 0.8 nm, and the spectra were recorded in the range from 200 to 1100 nm. A combined deuterium-tungsten reference light source was used to determine the spectral response of the spectrometer. The measured OES spectra were calibrated with this spectral response.

### 2.3. Surface Characterization

Approximately 20 min after plasma treatment, the surface composition of the samples was analysed by X-ray photoelectron spectroscopy (XPS). An XPS instrument model TFA XPS from Physical Electronics (Munich, Germany) was used. The samples were excited using monochromatic Al Kα_1,2_ radiation at 1486.6 eV. Photoelectrons were detected at an angle of 45° with respect to the normal of the sample surface. XPS survey spectra were measured at a pass-energy of 187 eV using an energy step of 0.4 eV. High-resolution C 1s spectra were measured at a pass-energy of 23.5 eV using an energy step of 0.1 eV. An additional electron gun was used for the surface charge compensation. All spectra were referenced to the main C 1s peak with a position set to 284.8 eV. The measured spectra were evaluated using MultiPak v8.1c software (Ulvac-Phi, Inc., Kanagawa, Japan, 2006) from Physical Electronics.

The surface wettability was measured 5 min after plasma treatment by a See System (Advex Instruments, Brno, Czech Republic). Contact angles (WCA) were determined with a demineralized water droplet of a volume of 3 μL. Three measurements were taken to minimize the statistical error.

The surface roughness and morphology were analysed by atomic force microscopy (AFM) using a Solver PRO (NT-MDT, Moscow, Russia) in tapping mode. The surface roughness, R_a_, was measured over an area of 5 μm × 5 μm.

## 3. Results and Discussion

**Figure 2:**
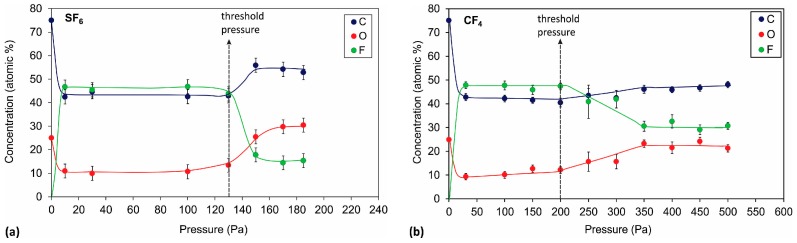
Surface composition of the PET samples treated at various pressures, as determined by XPS: (**a**) treated in SF_6_ plasma and (**b**) treated in CF_4_ plasma. Two different regions regarding the surface composition are observed at low/high pressures. The values of the atom concentration at a pressure of 0 Pa correspond to the untreated sample.

Figure 2a shows the variation of the XPS surface composition of the PET polymer treated in SF_6_ plasma versus pressure. The values for the atomic concentration at a pressure of 0 Pa correspond to an untreated sample. The measured values for the untreated sample, i.e., 25 at % oxygen and 75 at % carbon, are close to the theoretical values for pure polyethylene terephthalate. These values are altered upon plasma treatment, as demonstrated in Figure 2a.

As expected, fluorine appeared on the surface, and its concentration was dependent upon the pressure in the discharge chamber during the plasma treatment of the polymer sample. Two regions can be distinguished. The first one appeared at pressures up to approximately 130 Pa, where the surface composition was relatively constant and was independent of the pressure. However, at pressures higher than 130 Pa, a drastic (and rather abrupt) modification of the surface composition occurred. Hereinafter, a pressure of 130 Pa is considered the threshold pressure. In the first region, below the threshold pressure, plasma treatment resulted in intensive fluorination of the polymer because a high fluorine content of approximately 46 at % was found. Furthermore, the oxygen concentration decreased from the initial 26 to 10 at %. In the second region, above the threshold pressure, the fluorine concentration on the polymer surface dropped to only ~ 16 at %, whereas the oxygen concentration increased to almost 30 at %. Another important difference in both regions was the presence of a minor concentration of other elements. In addition to carbon and oxygen, which were already present in the original polymer, only fluorine was found in the first region. However, in the second region, a minor concentration of sulphur from SF*_x_* radicals was found as well (<1 at %). A detailed reason for this transition will be explained later in the text. It is correlated with the concentration of F atoms in the plasma, which was lower after the threshold pressure; therefore, surface fluorination was less efficient. Furthermore, all vacuum systems contain water vapour, which dissociates to O and OH radicals that compete with F atoms and cause oxidation. For this reason, a higher oxygen concentration was found above the threshold pressure. This phenomenon, whereby oxidation may occur when treating materials in F-containing plasmas, has been observed before and was published in [19].

**Figure 3:**
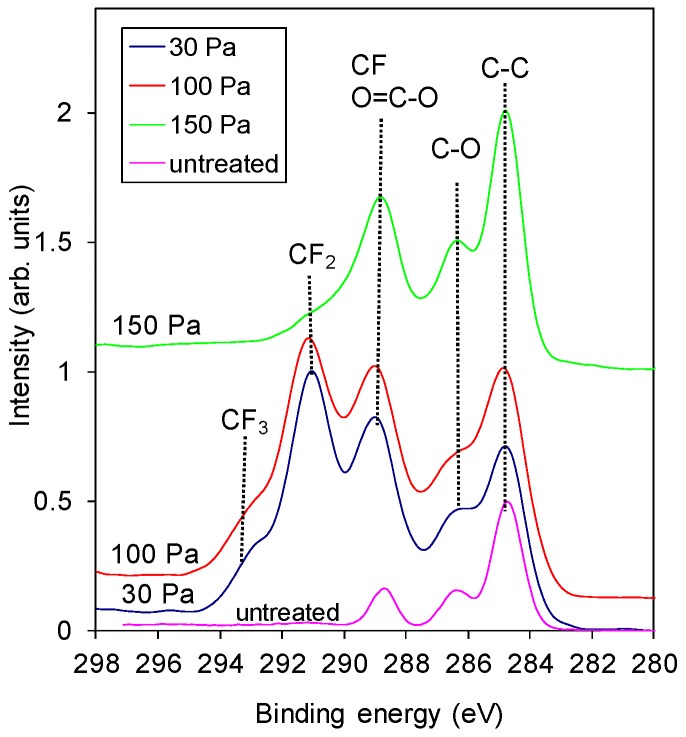
Comparison of high-resolution spectra C 1s of samples treated at various SF_6_ pressures. A spectrum for the untreated PET is added for comparison.

**Figure 4:**
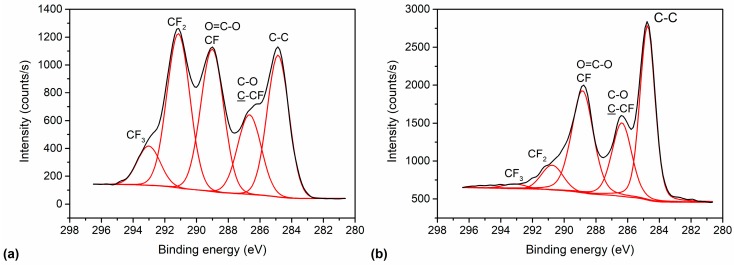
Deconvolution of C 1s spectra: (**a**) below and (**b**) above the threshold pressure.

The significant change in the surface concentration of F and O before and after the threshold pressure is also observed in the high-resolution carbon C 1s spectra shown in Figure 3. The samples treated at pressures below the threshold pressure were rich in CF_3_ and CF_2_ as well as CF functional groups (see Figure 4 also), whereas the sample treated at pressures higher than the threshold pressure had only some CF groups and an insignificant number of CF_2_ functional groups in the surface film probed by the photoelectrons. This result is shown in more detail in Figure 4, where an example of a detailed curve deconvolution of the C 1s spectra showing peak assignment is presented. The C 1s peak was fitted with five components positioned at the binding energies of 284.8 eV assigned to C–C, 286.5 eV assigned to C–O and C–CF, 289 eV assigned to O–C=O and CF, 291.3 eV to CF_2_ and 293 eV to CF_3_ [20,21]. 

**Figure 5:**
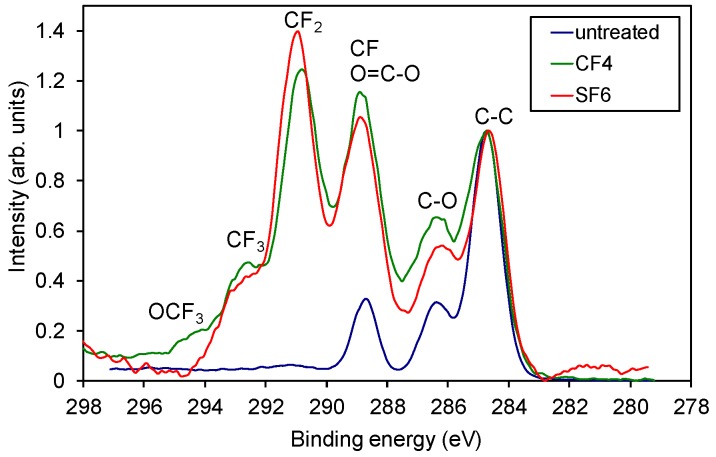
Comparison of selected high-resolution C 1s spectra of the untreated PET sample and the plasma-treated samples in the first region, below the threshold pressure.

When treating the polymer in CF_4_ plasma (Figure 2b), a similar behaviour was observed as when treated in SF_6_ plasma. However, the transition between the regions of high and low fluorine content was not very sharp (it appeared approximately at a threshold pressure of 200 Pa) and was less intense (the fluorine concentration dropped to only 30 at %, and oxygen increased to almost 23 at %). In the first region below the threshold pressure, there was no significant difference in the fluorine concentration on the sample treated in CF_4_ or SF_6_ plasma, (Figure 2a,b, respectively). Figure 5 shows a comparison of selected carbon peaks for the samples treated in CF_4_ and SF_6_ plasma at 100 Pa (low-pressure region). We see only minor differences in the intensity of the various fluorine functional groups of CF, CF_2_, and CF_3_ and the presence of OCF_3_ at ~295 eV for the sample treated in CF_4_ plasma.

**Figure 6:**
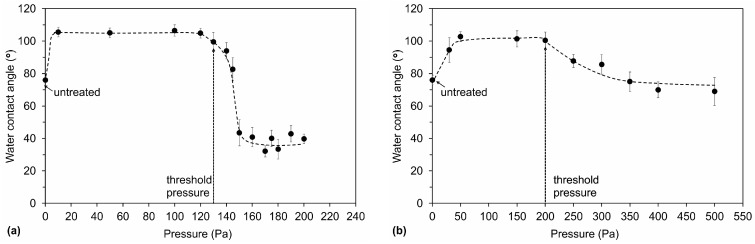
Water contact angles for the samples treated in: (**a**) SF_6_ plasma and (**b**) CF_4_ plasma at various pressures.

Plasma treatment changed the surface hydrophobicity of the samples. The water contact angle increased from the initial 76° to approximately 106° regardless of using the CF_4_ or SF_6_ plasma as long as the pressure was low enough (below the threshold). The value of 106° is typical for hydrophobic materials with a smooth surface [22]. The variation of the contact angle with pressure is interesting, as shown in Figure 6a. We can see that after the threshold pressure, when a decrease in fluorine and an increase in the oxygen concentration were observed (Figure 2a), the water contact angle significantly decreased to approximately 35°. The surface lost its hydrophobic character and became hydrophilic because of a lack of nonpolar fluorine functional groups and the presence of more polar oxygen groups. Interestingly, the water contact angle for PET treated at high pressure in SF_6_ plasma was much lower than that for the untreated polymer, which was 76°. Measurements of the surface roughness by AFM showed only a slight increase in the roughness from 1.2 nm measured for the untreated sample to 2.3 and 2.6 nm measured for the samples treated in SF_6_ and CF_4_ plasma, respectively. Therefore, only a minor influence of the surface roughness on the contact angles was observed, and the major reason for modified wettability is thus chemical modification of the surface. Figure 6b also shows the results for the CF_4_ plasma. Similar to the results obtained for SF_6_ plasma treatment, we observed that, after the threshold pressure, the contact angle decreased. However, the decrease was less pronounced, which is correlated with a lower oxygen content in comparison to the sample treated in SF_6_ plasma (Figure 2).

**Figure 7:**
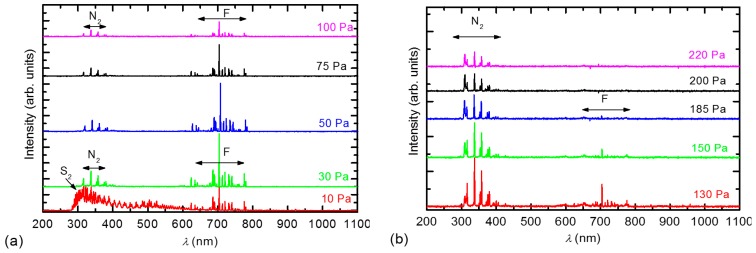
OES spectra of SF_6_ plasma at various pressures: (**a**) below the threshold pressure and (**b**) above the threshold pressure.

To explain such unusual behaviour of the surface composition and surface wettability with the pressure, we performed OES characterization of the plasma. OES spectra are shown in Figure 7. Figure 7a shows the spectra measured at low pressures (before the threshold pressure), while Figure 7b shows the spectra measured above the threshold pressure.

**Figure 8:**
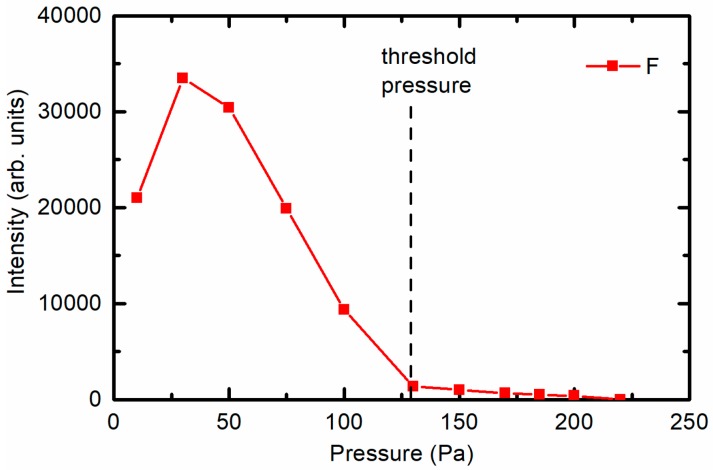
OES intensity of the F emission line at 703 nm versus SF_6_ pressure.

At low pressures, intensive atomic F lines in addition to bands corresponding to the N_2_ molecule are observed (Figure 7a). One exception is the spectrum measured at the lowest pressure 10 Pa, where bands corresponding to the S_2_ molecule are observed as well. The appearance of the S_2_ molecules can only be explained by the almost full dissociation of SF_6_ and the subsequent recombination to sulphur dimers. The presence of nitrogen, which is known to be a strong emitter, was explained as an impurity present in the original gas according to the manufacturer’s specifications. At high pressures (Figure 7b), the situation was different because the intensity of the F lines decreased. The variation in the F emission intensity with pressure is plotted in Figure 8. Figure 8 is in excellent agreement with Figure 2; at low pressures, where the emission intensity of F is high, the concentration of fluorine on the polymer surface is high. Whereas at higher pressures, when the OES intensity of F decreased, the XPS concentration of fluorine decreased.

This phenomenon deserves further discussion. In SF_6_ plasma, SF_x_ dissociates according to [23,24]:

(1) $$\begin{array}{c} SF_{x}+e^{-}\to SF_{x-1}+F+e^{-},\\ x=1–6,\;W_{D}=9.6\;eV \end{array}$$

The extent of dissociation and thus the concentration of radicals such as SF_5_, SF_4_, SF_3_, SF_2_, SF, S and F depends on the electron density and temperature, which in turn depends on the pressure. According to Kokkoris et al., a loss of SF*_x_* and F species on the reactor walls is also important for the production and consumption of neutral plasma species [23]. The F atoms tend to associate with F_2_ molecules either by heterogeneous surface recombination or in the gas-phase—the probability of gas-phase loss increases as a square of the pressure, because three-body collisions are necessary.

SF_6_ gas is also known to be a highly electronegative gas, which means that it has a strong tendency to acquire free electrons, thus forming negative ions: $e^{-}+SF_{6}\to SF_{6}^{-}$ [24,25]. At low pressures, the electron temperature is high, thus causing a strong dissociation of SF_6_ and, thus, the occurrence of a high density of F atoms in the plasma. The F atoms diffuse and eventually reach the polymer surface where they interact chemically and cause at least partial substitution of oxygen in the surface film of the PET polymer. The exact interaction mechanism is still unknown, but a high fluence of F atoms onto the surface of the polymer will guarantee the substitution of almost all oxygen in the PET surface by fluorine. The curves in Figure 3 obtained at 30 and 100 Pa confirm this simplified explanation.

At high pressures, however, the electron density and temperature decrease; therefore, the dissociation of SF_6_, which has a relatively high dissociation energy of *W*_D_ = 9.6 eV, is less effective [24]. Furthermore, the loss of F atoms due to the gas phase reactions becomes important. Electrons are also lost by the attachment to SF_6_ molecules. The lack of electrons capable of SF_6_ dissociation caused the density of the fluorine atoms in the plasma to decrease significantly at elevated pressure.

**Figure 9:**
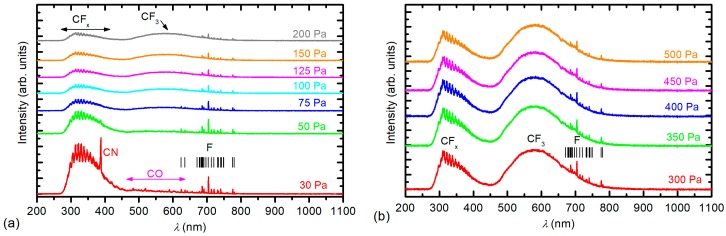
OES spectra of CF_4_ plasma at various pressures: (**a**) below the threshold pressure and (**b**) above the threshold pressure.

Similar conclusions can be drawn for SF_6_ plasma as for CF_4_ plasma. However, CF_4_ is as strongly electronegative as SF_6_; therefore, this effect is not very pronounced. For the CF_4_ plasma, F atomic lines are observed in the OES spectra at low pressures up to the threshold pressure (Figure 9a). The CF_3_ continuum is not observed below the threshold pressure (200 Pa), where it is barely noticeable. 

Therefore, we can expect good dissociation of CF_4_ at low pressures according to [26]:

(2) $$\begin{array}{c} CF_{x}+e^{-}\to CF_{x-1}+F+e^{-},\\ x=2–4,\;W_{D}=12.5\;eV \end{array}$$

**Figure 10:**
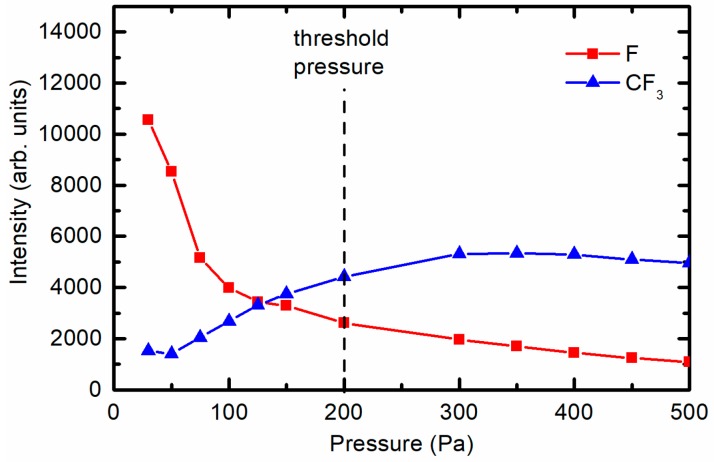
OES intensity of the F emission line at 703 nm and the CF_3_ band at 580 nm versus CF_4_ pressure.

The intensity of the F line decreased with increasing pressure. Furthermore, a continuum corresponding to CF_3_ appeared at high pressures (Figure 9b). The appearance of the CF_3_ continuum coincided with a decrease in the F intensity. Figure 10 represents the radiation intensity arising from the F atoms and the CF_3_ radicals. At elevated pressure, the radiation from the F-atoms became marginal, indicating qualitatively that the dissociation of multiple CF_4_ molecules was scarce. Comparing Figure 2 and Figure 10, we can again conclude that at high pressures, the electron temperature and density was so low that it was insufficient to cause substantial dissociation of CF_4_, and thus, the substitution of oxygen with fluorine on the PET polymer upon plasma treatment was poor.

## 4. Conclusions

Fluorination of the polymer surface in SF_6_ and CF_4_ plasma was investigated. Plasma was created at various pressures. It was observed that at low pressures up to the threshold pressure, the XPS concentration of fluorine on the polymer surface was high (~46 at %) regardless of the gas used. After the threshold pressure, a sudden decrease in the fluorine concentration was observed, which was more pronounced for the SF_6_ plasma. Simultaneously, the concentration of oxygen increased. Therefore, the surface changed from hydrophobic to hydrophilic. The threshold pressure for the SF_6_ plasma was ~130 Pa, whereas for the CF_4_ plasma it was slightly higher at ~200 Pa. This effect was explained by the electronegativity of both gases, especially SF_6_. At low pressures up to the threshold pressure, electrons can cause the full dissociation of gas molecules in plasma giving rise to a high concentration of fluorine radicals, which are responsible for surface fluorination. Because the density of fluorine in the plasma was high, the surface was fully saturated with the fluorine functional groups. At high pressures, the electron density and temperature decreased. Furthermore, they were also lost by electron attachment; therefore, the gas dissociation was weak, thus causing poor surface reactions.
